# The Association between Smoking and Epiretinal Membrane

**DOI:** 10.1038/srep38038

**Published:** 2016-11-29

**Authors:** Sheng-Zhan Wang, Qi-Hu Tong, Hui-Yun Wang, Qin-Kang Lu, Yu-feng Xu

**Affiliations:** 1 Ningbo Optometry & Ophthalmology Center, the Affiliated Eye Hospital of Wenzhou Medical College, Ningbo Yinzhou people’s Hospital, Ningbo, Zhejiang, 315040, China; 2 Department of Ophthalmology, the Second Affiliated Hospital of Zhejiang University, College of Medicine, Jiefang Road 88, Hangzhou, Zhejiang, 310009, China

## Abstract

We conducted a meta-analysis of analytic and observational studies to evaluate the association between smoking and epiretinal membrane (ERM). The pertinent studies were identified via a literature search using three databases (MEDLINE, Cochrane Library, Embase) and the reference lists of retrieved studies. Cohort, case-control and cross-sectional studies meeting the predefined criteria were included. We extracted the odds ratios (OR) and 95% confidence intervals (CI) from each study. Overall risk estimates were pooled using random-effects models. Subgroup analyses based on several stratified factors were also performed. Two cohort studies and six cross-sectional studies involving 46,837 subjects were included. The pooled effect of all eight studies showed an unexpected significant decreased association between smoking and the occurrence of ERM (OR, 0.72; 95% CI 0.61–0.84; p = 0.29, I^2^ = 17.9%). Subgroup analyses supported this finding, except for the age-unadjusted group (OR, 0.87; 95% CI 0.63–1.22), the ERM classification group (cellophane macular reflex (CMR) OR, 0.93; 95% CI 0.68–1.28; preretinal macular fibrosis (PMF) OR, 0.74; 95% CI 0.41–1.32), the Asian group (OR, 0.75; 95% CI 0.52–1.09) and the past smoker group (OR, 1.02; 95% CI 0.85–1.22). The pooled effects from the current literature suggested a declining association between smoking and ERM, which requires further studies to confirm.

Epiretinal membrane (ERM) is a common retinal condition that typically occurs in people aged 50 years and older and is characterized by the proliferation of abnormal tissues on the inner retinal surface^1^,^2^, which results in mild to moderate visual impairment with an impact on quality of life^3^. Electron microscopy has shown glial cells, retinal pigment epithelium (RPE) cells, fibrocytes, myofibroblasts, and fibrous astrocytes to be involved^4^. The early form of ERM, in which there is a patch or patches of irregular increased reflection from the inner retinal surface is sometimes called cellophane macular reflex (CMR) and is usually asymptomatic. Meanwhile, the more severe form, which includes surface wrinkling retinopathy and is termed preretinal macular fibrosis (PMF)^5^,^6^, can cause significant loss of visual acuity and visual symptoms via tangential traction in the macula area^7^.

In recent years, studies among different ethnic groups have provided population-based prevalence data on ERM, ranging from 1.0% to 28.9%^5^,^8^,^9^,^10^,^11^,^12^,^13^,^14^,^15^,^16^,^17^,^18^. Despite much effort in epidemiologic, clinical and laboratory research, the pathogenesis and risk factors for ERM remain incompletely understood. There is still a great discrepancy in existing studies regarding possible risk factors, other than age, for primary ERM^5^,^11^,^17^.

It is know that ERM formation is a fibrotic process that occurs on the surface of the retina^1^, with most ERMs containing fibroblasts and myofibroblasts^4^,^19^. Evidence has shown that retinal Müller cells, hyalocytes, and RPE cells have the ability to differentiate into a myofibroblastlike phenotype that is responsible for excessive collagen production and deposition, as well as the contractile activity of ERMs^20^,^21^,^22^. The retinal (outer) side of the ERM usually consists of an extracellular matrix (ECM) layer containing bundles of extracellular fibrils, which are usually randomly oriented. The exact origin of the collagenous components in the ECM of ERM remains unclear^1^. Meanwhile, smoking is a well-known risk factor for a wide range of diseases, including vascular disease, lung cancer, and chronic obstructive pulmonary disease^23^,^24^,^25^. Additionally, smoking-associated fibrosis has recently been described by several researchers^26^,^27^. Checa *et al.*^28^ demonstrated that cigarette smoke extract induced the expression of a variety of profibrotic genes, and the activation of TGF-β1, resulting in the excessive accumulation of ECM and the activation of myofibroblasts, which also plays a crucial role in the pathogenesis of ERM^1^. Thus, it is of great interest to examine whether smoking behavior is related to ERM.

## Methods

### Search Strategy

We followed the MOOSE checklist to conduct this meta-analysis. (see Supplementary Material). We conducted a systematic search through three databases, including MEDLINE (1950 to October 2015), the Cochrane Library (up to October 2015), and Embase (1980 to October 2015), using the following MeSH terms and keywords: smoking OR tobacco OR cigarette OR lifestyle OR risk factor OR epidemiology, combined with epiretinal membrane OR cellophane macular reflex OR preretinal macular fibrosis. References in the retrieved publications were reviewed to identify other pertinent studies. No language restrictions were imposed.

### Study Selection

Studies were considered eligible if they met the following criteria: (1) they were cohort or case–control or cross-sectional studies published as original articles; (2) they estimated the relationship between smoking and the risk of ERM with the odds ratio (OR) and 95% confidence interval (CI) or provided any other calculable information; and (3) they defined OR of smoking as current smoker VS none/never smoker or current smoker VS none/never or past/former/ever smoker. When articles with overlapping participants were available, we selected the most recent study. We contacted the authors of related articles for necessary data such as ORs of association between smoking and ERM and ORs concerning smoking status and other stratified factors to determine whether the article met our inclusion criteria. We excluded the literature sources such as conference abstracts, as they lacked adequate information for evaluation of their validity and reliability.

Two of the authors (Q-HT and S-ZW) reviewed the titles and abstracts independently to exclude any obviously irrelevant studies. If uncertainty regarding suitability remained, full manuscripts were subsequently obtained. Any disagreement was resolved by discussion.

### Data Extraction and Study Quality Evaluation

For each study, the following characteristics were extracted: (1) last name of first author, (2) publication year, (3) country in which the study was conducted, (4) study design, (5) study period, (6) population type and sample size, (7) mean age or age range of study subjects, (8) smoking status, (9) ERM classification, (10) ERM definition and grating, (11) ERM prevalence and (12) adjusted variables. Methodological quality was assessed using the validated Newcastle–Ottawa quality assessment scale (for cohort studies)^29^ and the Cross-Sectional Study Quality scale (for cross-sectional studies)^30^.

### Data Synthesis

The association between smoking and ERM was evaluated by calculating pooled ORs and 95% CIs. We used age-adjusted or gender-adjusted ORs whenever possible. However, several articles did not provide adjusted ORs (shown in Table 1), in which case we used the unadjusted ORs. Smoking was defined as current smoker VS none/never smoker or current smoker VS none/never or past/ever smoker, except for the subgroup analysis of smoking status where smoking is defined as current smoker VS past smoker. The significance of the pooled OR was determined by Z test (P < 0.05 was considered statistically significant, unless otherwise specified). ERMs were classified as either early or late stage, commonly corresponding to CMR or PMF, respectively. Subgroup analysis was performed based on study design, subject race, smoking status, ERM classification and age adjustment. In addition, sensitivity analysis was also performed.

Heterogeneity among the included studies was tested the Cochran Q and I^2^ statistics^31^. Significant heterogeneity was detected if the P value was <0.1 or I^2^ was >50%^32^. According to the Cochrane Reviewers’ Handbook Version 4.2.2, results from random-effects model are more conservative and they approximate results more precisely from the fixed-effects model when no significant heterogeneity is detected, so we used a random-effects model throughout all analyses. Potential publication bias was assessed by Egger’s test^33^ and Begg’s test^34^. A sensitivity analysis, which removed each study one at a time, was conducted to confirm the stability of the findings. All statistical analyses were performed using commercially available software (STATA 12.0; StataCorp LP). The level of significance was set to P < 0.05, unless otherwise specified.

## Results

### Identification and Selection of Studies

**Figure 1:**
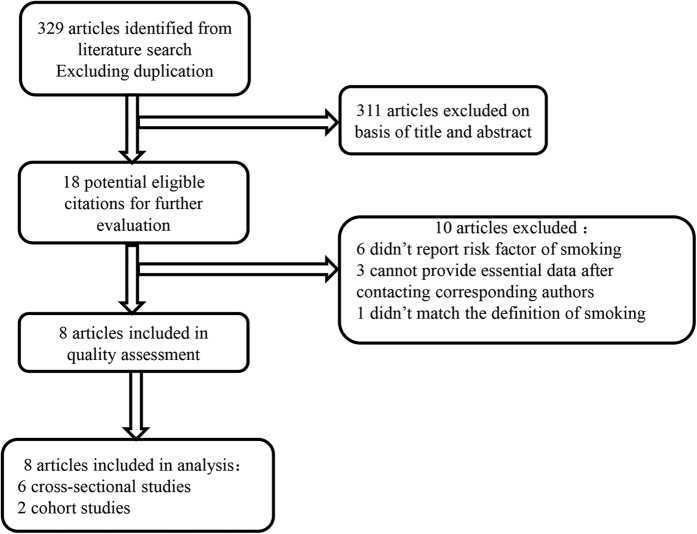
Flow chart showing the study selection process.

Our search of three databases identified 329 articles. After screening of the titles and abstracts, 311 unrelated articles were excluded as irrelevant. In further evaluation of the remaining 18 articles, 10 were excluded for the following reasons: 6^13^,^14^,^35^,^36^,^37^,^38^ did not report the risk factor of smoking; 3^5^,^11^,^39^ could not provide essential data after contacting the corresponding authors and 1^15^ did not meet the definition of smoking. In the end, two cohort studies^10^,^18^ and six cross-sectional studies^9^,^12^,^16^,^17^,^40^,^41^ met our criteria (Fig. 1).

### Study Characteristics and Study Quality Evaluation

The detailed characteristics of the eight selected studies are summarized in Table 1. The eight studies involved 46,837 participants from 1992 to 2013. In terms of the participants’ ethnicities, four studies were conducted on Asian populations, three were conducted among Caucasians and one was a multi-ethnic study. All the studies were volunteers-based or population-based. Participants in all studies were 40 years of age or older. Smoking status was mainly divided into “never/none”, “past/former/ever” and “current”. ERM classification mostly involved “any ERM”, “CMR” and “PMF”. Various diagnostic methods (e.g., fundus photography, optical coherence tomography (OCT) or indirect ophthalmoscopy) were used. And the prevalence of any ERM in different studies ranged from 3.4% to 28.9%. Several studies provided age, gender or other variable-adjusted ORs, while others did not. Study quality evaluation can be seen in the supplemental materials. (Table S1 for cross-sectional studies, Table S2 for cohort studies).

### Association Between Smoking and ERM

**Figure 2:**
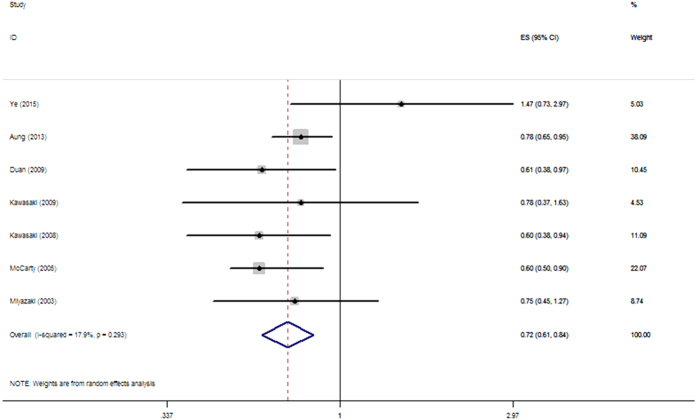
Pooled estimates of the association between smoking and ERM in total analysis. OR, odds ratio, CI, confidence interval.

In the overall analysis, we detected that smoking was associated with a significantly decreased risk of ERM (OR, 0.72; 95% CI 0.61–0.84) without significant heterogeneity (P = 0.293, I^2^ = 17.9%) (Fig. 2). We then conducted subgroup analyses based on the following stratified factors:

1. Study design: In the study-specific analysis, significant association was also identified in the cohort group (OR, 0.78; 95% CI 0.65–0.94; P = 1.00, I^2^ = 0.0%) and cross-sectional group (OR, 0.69; 95% CI 0.54–0.88; P = 0.206, I^2^ = 32.4%) (Figure S1).
2. Population ethnicity: In consideration of difference in hereditary susceptibility or environmental factors, we pooled estimates based on ethnicity. Although smoking was not associated with ERM in Asians (OR, 0.75; 95% CI 0.52–1.09; P = 0.163, I^2^ = 41.4%), a significantly decreased risk between smoking and ERM was found in Caucasians (OR, 0.72; 95% CI 0.61–0.85; P = 0.336, I^2^ = 8.2%) (Figure S2).
3. Smoking status: We also sought to investigate whether smoking has a lasting impact on ERM pathology. To this end, comparisons between past smokers or current smokers and those who had never smoked were undertaken. Current smokers displayed a significantly decreased risk of ERM (OR, 0.73; 95% CI 0.62–0.86; P = 0.416, I^2^ = 0.0%), while a nonsignificant relationship was found in past smokers (OR, 1.02; 95% CI 0.85–1.22; P = 0.285, I^2^ = 20.4%) (Figure S3).
4. ERM classification: We pooled estimates in terms of different stages of ERM, although no significant correlation with smoking was revealed in the early-stage group (CMR) (OR, 0.93; 95% CI 0.68–1.28; P = 0.041, I^2^ = 68.7%) or in late-stage group (PMF) (OR, 0.74; 95% CI 0.41–1.32; P = 0.007, I^2^ = 68.9%) (Figure S4).
5. Adjustment for age: Increasing age has been consistently demonstrated to be a risk factor for ERM, we assessed the outcomes in both the age-adjusted group (OR, 0.62; 95% CI 0.51–0.76; P = 0.899, I^2^ = 0.0%) and the group not adjusted for age (OR, 0.87; 95% CI 0.63–1.22; P = 0.231, I^2^ = 31.8%) (Figure S5).

### Publication Bias and Sensitivity Analysis Study

**Figure 3:**
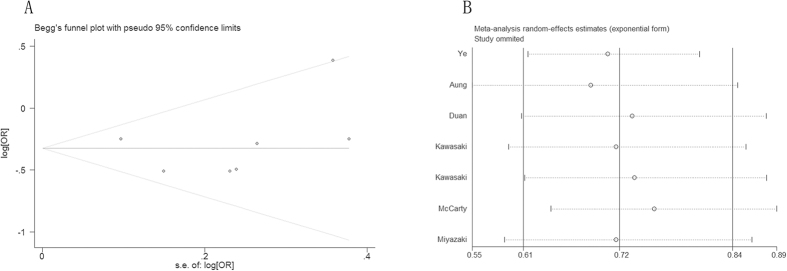
Funnel plots chart according to Begg’s rank correlation test and sensitivity analysis.

Begg’s rank correlation test and Egger’s linear regression test indicated no evidence of significant publication bias among the included studies (Begg, P = 0.23; Egger, P = 0.815), and funnel plots showed a symmetric distribution (Fig. 3A). Sensitivity analysis showed no significant changes after removing one study at a time, indicating the stability of these findings (Fig. 3B).

## Discussion

Our meta-analysis showed that there was a significant decreasing association between smoking behavior and risk of ERM in cohort and cross-sectional studies. Similar significant relationships were also found in the stratified analyses except for ERM classification, Asian ethnicity, past smoker status and age-unadjusted subgroups.

ERM consists of a sheet of fibrotic tissue that varies in thickness from a single layer of collagen with interspersed cells to a thicker, multilayered fibrocellular proliferation^42^,^43^. Although the pathogenesis of ERM has not yet been fully elucidated, the formation and progression of ERM can be regarded as a fibrotic process because the pathologic findings include increased ECM protein deposition and membrane contraction in which myofibroblasts play a crucial role^1^. Several studies have demonstrated that exposure to cigarette smoke extracts can provoke activation of the TGF β pathway and the epithelial-mesenchymal transition (EMT)^44^,^45^, and thereby up-regulate numerous genes involved in fibrosis and the accumulate of ECM^28^ and these processes are also known to plays a crucial roles in the pathogenesis of ERM. However, the results of epidemiologic studies of the relationship between smoking and risk of ERM have remained inconclusive.

The unexpected protective association between smoking and ERM was also observed in other studies^5^,^15^,^16^,^40^. Although no adequate explanation has been provided, some researchers have suggested several possible reasons for this finding. Kawasaki *et al.*^16^ attributed this unexpected protective association to the substantial difference in the proportion of male and female current smokers in their study participants. As a result, 92.7% of current smokers were men. In addition, current smokers were significantly younger than non-smokers or past smokers. After adjusting for age and gender, the inverse association between current smoking and ERM was attenuated (unadjusted OR 0.39 (CI 0.26 to 0.59); age–gender-adjusted OR 0.60 (CI 0.38 to 0.94)). However, residual influence from the substantial gender difference in the proportion of current smokers cannot be excluded. Another explanation proposed by McCarty *et al.*^40^ was a survival effect caused by higher mortality among male current smokers. And in addition, Duan *et al.*^9^ have demonstrated that neither the number of cigarettes smoked per day nor smoking years was significantly associated with ERMs, suggesting that this negative association with smoking could have been a spurious finding or could have been due to decreased survival among smokers. Thus, no consensus exists, and further evidence is needed.

Studies have reported significant discrepancy in the prevalence of ERM among different ethnic groups. Some studies have indicated that the prevalence of ERM in the Caucasian population is much higher than in the Asian population^9^,^13^,^14^. In our meta-analysis, we also discovered different associations among ethnic groups with smoking behavior. The underlying explanation for these racial/ethnic variations is unclear. One reason for the discrepancies between studies may be ethnic differences in the background pigmentation of the fundus, leading to differences in the ophthalmoscopic visibility of the ERM^13^. Meanwhile, this discrepancy could also be attributed to systematic differences in the definition and detection methods of CMR and PMF as well as differences in the grading of these retinal conditions according to Kawasaki *et al.* Another intriguing possibility is the higher rates of smoking among Asians. Indeed, it has been reported that smoking rates are higher in China and Japan than in Australia and the United States^46^,^47^,^48^,^49^. Owing to the apparent inverse association between smoking and ERMs, the higher prevalence of cigarette smoking may explain the lower rates of ERMs in our study^9^. However, such a hypothesis remains to be verified.

We also detected a bit smaller ORs of age-adjusted studies than age-unadjusted studies. Several studies have observed the reverse associations between current smoking and ERM, which they tended to explain by age constitution and survival effect. And the reverse association was attenuated after adjusting age in the research of Kawasaki *et al.*^16^, which is different from our subgroup analysis. If the hypothesis was real, it’s reasonable to assume the adjusted ORs would decrease, compared with unadjusted ORs within same study. However, among different studies and combined effects, it’s still so complicated to reach such a conclusion that adjusted one would be attenuated. One possible explanation is the heterogeneities of methodologies, subject ethnicities and statistical analyses between different studies. Although, P values and I^2^ didn’t show statistical significance in most situations, they did exist and could affect the results in some degree. Considering few studies provided both age-adjusted and age-unadjusted ORs, we could neither test the hypothesis nor simply compare the absolute values of ORs among studies and combined effects.

Several limitations in our work cannot be ignored. First, there were only eight studies with two different study designs included in our meta-analysis. However, the heterogeneity of total pooled estimates was not significant, which indicated good homogeneity among the included studies. Moreover, sensitivity analysis showed no significant changes after removing one study at a time, indicating the stability of these findings. Still, we should be cautious about generalizing our conclusions to other conditions. Secondly, we defined ORs of smoking as current smoker VS none/never smoker or current smoker VS none/never or past/former/ever smoker, which arbitrarily combined the effects of none smoker and past smoker. Coincidentally, subgroup analysis based on smoking status supported our assumption that differences between the two groups were not significant. Self-reported questionnaires and medical records were widely adopted in these including articles, so unclearly definition of smoking status and recall bias could affect the association. While, obscure smoking status definition of McCarty *et al.* may also attenuate the difference between none smoker and past smoker. Still, we can not get to a conclusion whether it is a fact or statistical spuriousness. Third, the participants were population-based or volunteer-based. It is a common view that volunteers might be more conscientious about maintaining a healthy lifestyle, which could dilute the observed association. Fourth, we failed to evaluate the dosage– response association owing to insufficient data on smoking consumption and different durations. Furthermore, the study of McCarty *et al.*^40^ did not clearly define smoking status, we contacted Dr. McCarty to confirm this definition, but received no reply. To take full use of the existing literature, we still included this study in our analysis. Despite the ambiguous definition, the sensitivity analysis showed an unchanged result, which reduced the risk.

In conclusion, this meta-analysis of analytic and observational studies revealed an unexpected beneficial effect of smoking related to ERM. We provide additional evidence on the association between smoking and ERM and also discuss the reason for this unusual association. Owing to the limitations of our work, we should be conservative in interpreting the pooled estimates. Further high-quality research that specifically adresses daily cigarette consumption and smoking duration is warranted to confirm our findings and provide a possible explanation for this association.

## Additional Information

**How to cite this article**: Wang, S.-Z. *et al.* The Association Between Smoking and Epiretinal Membrane. *Sci. Rep.* **6**, 38038; doi: 10.1038/srep38038 (2016).

**Publisher's note:** Springer Nature remains neutral with regard to jurisdictional claims in published maps and institutional affiliations.

## Supplementary Material

Supplementary Information

## Figures and Tables

**Table 1:** Summary of included studies evaluating smoking and its association with ERM.

Source (Publication Year,Country)	Design	Study Period	Population (Sample Size)	Age Range* (Yrs)	Smoking Status	ERM Classification	ERM definition and grating	ERM Prevalence (%, 95% CI)	Adjusted variables
Ye *et al.*^41^ (2015 China)	Cross-sectional	2012–2013	Jiangning Eye Study, population-based (N = 2,005)	≥50	Never; Past; Current	Any ERM CMR PMF	Fundus photography, OCT images	Any ERM 7.3 (6.2–8.5) CMR 4.3 (3.4–5.2) PMF 3.0 (2.3–3.8)	None
Aung *et al.*^18^ (2013 Australia)	Cohort	2003–2007	Melbourne Collaborative Cohort Study, volunteers (N = 21,241)	65.2 ± 5.8	Never; Former; Current	Any ERM CMR PMF	Retinal imges	Any ERM 8.9 (8.48–9.25) CMR 4.9 (4.64–5.22) PMF 3.9 (3.67–4.19)	None
Ng *et al.*^17^ (2011 USA)	Cross-sectional	2000–2002	Multi-Ethnic Study of Atherosclerosis, population-based (N = 5960)	62	Current; Never;	Any ERM CMR PMF	Digital non-stereoscopic fundus photographs	Any ERM 28.9 CMR 25.1 PMF 3.8	Age, gender, ethnicity, study center
Duan *et al.*^9^ (2009 China)	Cross-sectional	2006–2007	Handan Eye Study, population-based (N = 6,565)	51.7 ± 11.6	Never; Past; Current	Any ERM CMR PMF Unclassified (by OCT only)	Retinal photograph and/or OCT	Any ERM 3.4 (2.9–3.8) CMR 2.2 PMF 0.7 Unclassified 0.5 0.3–0.6	Age, sex
Kawasaki *et al.*^10^ (2009 Japan)	Cohort	2000–2002	Funagata study, population-based (N = 1723)	64.9	Never; Past; Current	Any ERM CMR PMF	Non-mydriatic fundus photographs	Any ERM 5.7 (4.5–7.0) CMR 4.1 (3.0–5.2) PMF 1.6 (0.9–2.3)	None
Kawasaki *et al.*^16^ (2008 Singapore)	Cross-sectional	2004–2006	Singapore Malay, population-based (N = 3,265)	40–80	Never; Past; Current	Any ERM CMR PMF	Fundus photograghs	Any ERM 7.9 (7.1–8.7) CMR 3.8 (3.3–4.4) PMF 4.1 (3.4–4.7)	Age, gender
McCarty *et al.*^40^ (2005 Australia)	Cross-sectional	1992–1997	Visual Impairment Project, population-based (N = 4,313)	60.1 ± 12.8	Smoking history (Not clearly defined)	Any ERM CMR PMF	Fundus photograghs	Any ERM 6.0 (5.2–6.7) CMR 4.8 (4.0–5.6) PMF 1.7 (1.2–2.3)	Age, sex
Miyazaki *et al.*^12^ (2003 Japan)	Cross-sectional	1998	Hisayama study, population-based (N = 1,765)	≥40	Current habitual user; Nonuser	Any ERM CMR PMF	Indirect ophthalmoscopy, slit lamp, and color fundus photographs	Any ERM 4.0 CMR NA PMF NA	Age

ERM, epiretinal Membrane, CMR, cellophane macular reflex, PMF, preretinal macular fibrosis. *As limited information was provided, we failed to display age of participants in a uniform way, we extracted age range or mean with or without Standard deviation.
