# Supplementary material for: The Association between Smoking and Epiretinal Membrane
Source: Sci Rep. 2016 Nov 29;6:38038. doi: 10.1038/srep38038 (PMC5126679; doi:10.1038/srep38038)
Supplement: Supplementary Information [file srep38038-s1.pdf]

The Association Between Smoking and Epiretinal Membrane

Sheng-Zhan Wang[1], Qi-Hu Tong[1], Hui-Yun Wang[1], Qin-Kang Lu[1], Yu-feng Xu[2]

1. Ningbo Optometry & phthalmology Center, the Affiliated Eye Hospital of Wenzhou Medical College, Ningbo Yinzhou people's Hospital, Ningbo, Zhejiang, 315040, China;

2. Department of Ophthalmology, the Second Affiliated Hospital of Zhejiang University, College of Medicine, Jiefang Road 88, Hangzhou, Zhejiang, 310009, China

Correspondence: Yu-feng Xu, department of Ophthalmology, the Second Affiliated Hospital of Zhejiang University, College of Medicine, Jiefang Road 88, Hangzhou, Zhejiang, 310009, China, Tel: +86-571-87783814, Fax: +86-571-87783814, E-mail: xuyufeng0216@163.com. Qin-Kang Lu, Ningbo Optometry & phthalmology Center, the Affiliated Eye Hospital of Wenzhou Medical College, Ningbo Yinzhou people's Hospital, Baizhang East Road 251, Jiangdong District, Ningbo, Zhejiang, 315040, China. Tel: +86-574-87017513, Fax: +86-574-87017513, E-mail: Luqinkang@163.com

MOOSE Checklist

Criteria		Brief description of how the criteria were handled in the meta-analysis
**Reporting of background should include**		
√	Problem definition	1. What is the epidemiology of epiretinal membrane?<br>2. From the existing literature, is there a worldwide consensus for OR (Odds ratio) concerning the association between prevalence of epiretinal membrane and risk fact of cigarette consumption?<br>3. Does OR of this association change among population race, smoking status, epiretinal membrane classification or methodology of studies?
√	Hypothesis statement	Smoking influence the risk of epiretinal membrane, which may have different performance based on population race, smoking status, epiretinal membrane classification or study methodology.
√	Description of study outcomes	Prevalence interval of epiretinal membrane;<br>ORs of association between smoking and epiretinal membrane based on different stratified analysis.
√	Type of exposure or intervention used	Smoking: current, past/former/ever; none/never
√	Type of study designs used	We included prospective cohort studies and cross-sectional studies.
√	Study population	We placed no restriction.
**Reporting of search strategy should include**		
√	Qualifications of searchers	The credentials of the two investigators S-ZW and Q-HT are indicated in the author list. The corresponding author Y-FX has published several meta-analysis articles in different journals focus on ophthalmology and neurology.
√	Search strategy, including time period included in the synthesis and keywords	PubMed from 1950 – October 2015<br>EMBASE from 1980 – October 2015<br>Cochrane Library up to October 2015<br>using the following MeSH terms and keywords: smoking OR tobacco OR cigarette OR lifestyle OR risk factor OR epidemiology, combined with epiretinal membrane OR cellophane macular reflex OR preretinal macular fibrosis
√	Databases and registries searched	PubMed, EMBASE and Cochrane
√	Search software used, name	We did not employ a search software.

	and version, including special features	PubMed was accessed from the National Library of Medicine (free online);<br>Medline and Embase are available on the OVID SP platform;<br>Cochrane Library is available on the Wiley Interscience platform.<br>EndNote was used to merge retrieved citations and eliminate duplications.
√	Use of hand searching	We hand-searched bibliographies of retrieved papers for additional references.
√	List of citations located and those excluded, including justifications	Details of the literature search process are outlined in the flow chart (Figure 1). The citation list is available in Table 1.
√	Method of addressing articles published in languages other than English	We placed no restrictions on language; local scientists fluent in the original language of the article were contacted for translation.
√	Method of handling abstracts and unpublished studies	We excluded the gray literature, such as conference abstracts, as they lacked adequate information for evaluation of their validity and reliability.
√	Description of any contact with authors	We contacted Dr. Bamini Gopinath, Dr. Rohit Varma and Dr. Tien Y. Wong for OR of smoking; we contacted Dr. McCarty for smoking definition; we contacted Dr. Wang for ORs of current smoking VS none or past smoker. But received no useful information.
**Reporting of methods should include**		
√	Description of relevance or appropriateness of studies assembled for assessing the hypothesis to be tested	Detailed inclusion and exclusion criteria were described in the Study Selection section of Methods in the manuscript.
√	Rationale for the selection and coding of data	Studies were included and excluded as per criteria outlined in Methods;<br>Study selection was performed by Q-HT and validated by S-ZW with full agreement;<br>Data extracted from each of the studies were relevant to the study design, study period, population characteristics, age range, exposure status, epiretinal membrane classification, epiretinal membrane definition and epiretinal membrane prevalence;<br>Several studies did not present the overall risk estimates of ERM but only showed results of bifurcation analyses (e.g., ERM classification or smoking status). We used a fixed-effects model to calculate a combined OR.

√	Assessment of confounding	We used age-adjusted or gender-adjusted ORs as possible as we can in the overall pooled estimates.
√	Assessment of study quality, including blinding of quality assessors; stratification or regression on possible predictors of study results	We used Newcastle–Ottawa quality assessment scale for cohort studies and Cross-Sectional Study Quality scale for cross-sectional studies to assess study quality.
√	Assessment of heterogeneity	Heterogeneity of the studies were explored within two types of study designs using Cochrane's Q test of heterogeneity and I^2 statistic that provides the relative amount of variance of the summary effect due to the between-study heterogeneity. Subgroup analyses were conducted, trying to identify the possible source of heterogeneity. Sensitivity test was made to validate the stability of pooled estimates with significant heterogeneity.
√	Description of statistical methods in sufficient detail to be replicated	Description of methods of meta-analyses, sensitivity analyses, subgroup analyses and assessment of publication bias are detailed in the methods.
√	Provision of appropriate tables and graphics	We included 1 table for the data extraction, 1 table (supplementary material) for quality assessment of cohort study, 1 table (supplementary material) for quality assessment of cross-sectional study, 1 figure for flow chart, 1figure for total pooled estimates, 1 figure for publication bias test and sensitivity test, 5 figures (supplementary material) for subgroup analyses based on age adjusted, study design, epiretinal membrane classification, population sample, human race and smoking status.
**Reporting of results should include**		
√	Graph summarizing individual study estimates and overall estimate	Figure 2
√	Table giving descriptive information for each study included	Table 1
√	Results of sensitivity testing	Figure 3
√	Indication of statistical uncertainty of findings	95% confidence intervals were presented with all summary estimates.
**Reporting of discussion should include**		
√	Quantitative assessment of	Publication bias was assessed using funnel plots,

	bias	Begg's and Eger's tests;<br>Sensitivity analyses indicate heterogeneity in strengths of the association due to most common biases in observational studies.
√	Justification for exclusion	Papers were excluded on the basis of failing to meet the inclusion criteria listed in Methods of manuscript. We did not systematically exclude any studies on the basis of language or study population size.
√	Assessment of quality of included studies	Results of quality assessment are showed in Table S1 and Table S2. We discussed the results of the sensitivity analyses, and potential reasons for the observed heterogeneity.
**Reporting of conclusions should include**		
√	Consideration of alternative explanations for observed results	We discussed that potential unmeasured confounders such as survival effect or different smoking prevalence in male and female have caused the decreasing risk.<br>We listed several limitations of our current work such as failing to do dose-effect analysis and significant heterogeneity.
√	Generalization of the conclusions	Our meta-analysis of analytic and observational studies revealed a declining association between smoking and EMR, which need further studies to confirm.
√	Guidelines for future research	We recommend future high quality studies with specific claiming daily cigarette consumption and smoking duration focus on the association between smoking and epiretinal membrane.
√	Disclosure of funding source	This study was supported by grant 201101C8000001 from the foundation for science and technology innovation in rural, Ningbo. The authors declare no conflicts of interest.

Table S1: Quality Assessment of Cross-sectional studies

**Item**	Ye,[41] 2105	Ng,[17] 2011	Duan,[9] 2009	Kawasaki,[16] 2008	McCarty,[40] 2005	Miyazaki,[12] 2003
1) Define the source of information (survey, record review)	Yes	Yes	Yes	Yes	Yes	Yes
2) List inclusion and exclusion criteria for exposed and unexposed subjects (cases and controls) or refer to previous publications	Yes	Yes	Yes	Yes	Yes	Yes
3) Indicate time period used for identifying patients	Yes	Yes	Yes	Yes	Yes	Yes
4) Indicate whether or not subjects were consecutive if not population-based	Yes	Yes	Yes	Yes	Yes	Yes
5) Indicate if evaluators of subjective components of study were masked to other aspects of the status of the participants	Yes	Yes	No	Unclear	Unclear	Yes
6) Describe any assessments undertaken for quality assurance purposes (e.g., test/retest of primary outcome measurements)	No	Unclear	Unclear	No	No	Unclear
7) Explain any patient exclusions from analysis	No	No	Yes	No	Yes	Yes
8) Describe how confounding was assessed and/or controlled.	Yes	Yes	Yes	Yes	Yes	Yes
9) If applicable, explain how missing data were handled in the analysis	Unclear	Unclear	Unclear	Unclear	Unclear	Unclear
10) Summarize patient response rates and completeness of data collection	Yes	Yes	Yes	Yes	Yes	Yes
11) Clarify what follow-up, if any, was expected and the percentage of patients for which incomplete data or follow-up was obtained	No	No	No	No	No	Yes

Table S2: Quality Assessment of Cohort Studies based on Newcastle-Ottawa Quality Assessment Scale

**Item**	**Score**	
	Aung,[18] 2013	Kawasaki,[10] 2009
**Selection**		
Representativeness of the exposed cohort	NA	*
Selection of the non exposed cohort	*	*
Ascertainment of exposure	NA	NA
Demonstration that outcome of interest was not present at start of study	*	*
**Comparability**		
Comparability of cohorts on the basis of the design or analysis	**	*
**Outcome**		
Assessment of outcome	*	*
Was follow-up long enough for outcomes to occur	*	*
Adequacy of follow up of cohorts	*	*

Note: A study can be awarded a maximum of one star for each numbered item within the Selection and Outcome categories. A maximum of two stars can be given for Comparability.

Figure S1

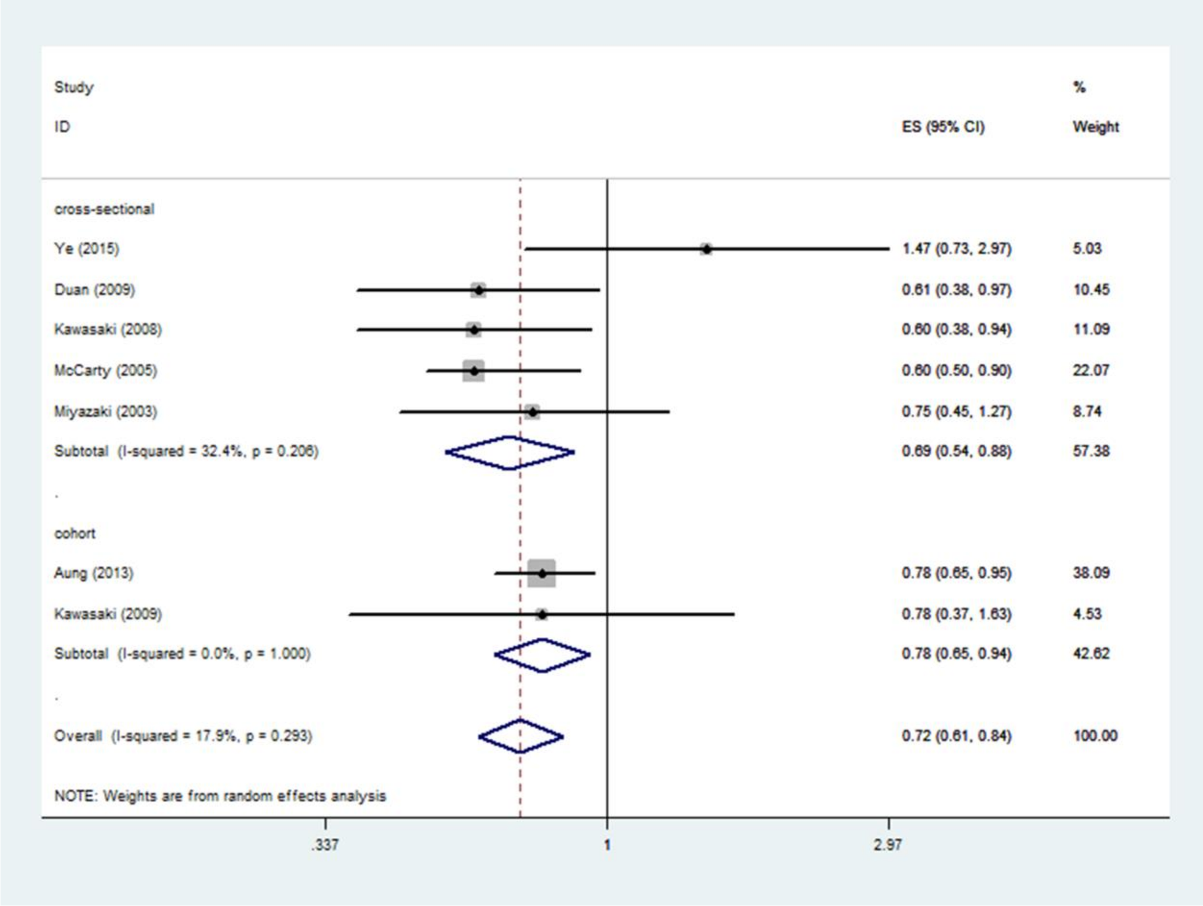

Study
ID
ES (95% CI)
%
Weight
cross-sectional
Ye (2015)
1.47 (0.73, 2.97)
5.03
Duan (2009)
0.61 (0.38, 0.97)
10.45
Kawasaki (2008)
0.60 (0.38, 0.94)
11.09
McCarty (2005)
0.60 (0.50, 0.90)
22.07
Miyazaki (2003)
0.75 (0.45, 1.27)
8.74
Subtotal (I-squared = 32.4%, p = 0.206)
0.69 (0.54, 0.88)
57.38
cohort
Aung (2013)
0.78 (0.65, 0.95)
38.09
Kawasaki (2009)
0.78 (0.37, 1.63)
4.53
Subtotal (I-squared = 0.0%, p = 1.000)
0.78 (0.65, 0.94)
42.62
Overall (I-squared = 17.9%, p = 0.293)
0.72 (0.61, 0.84)
100.00
NOTE: Weights are from random effects analysis
.337
1
2.97

Figure S2

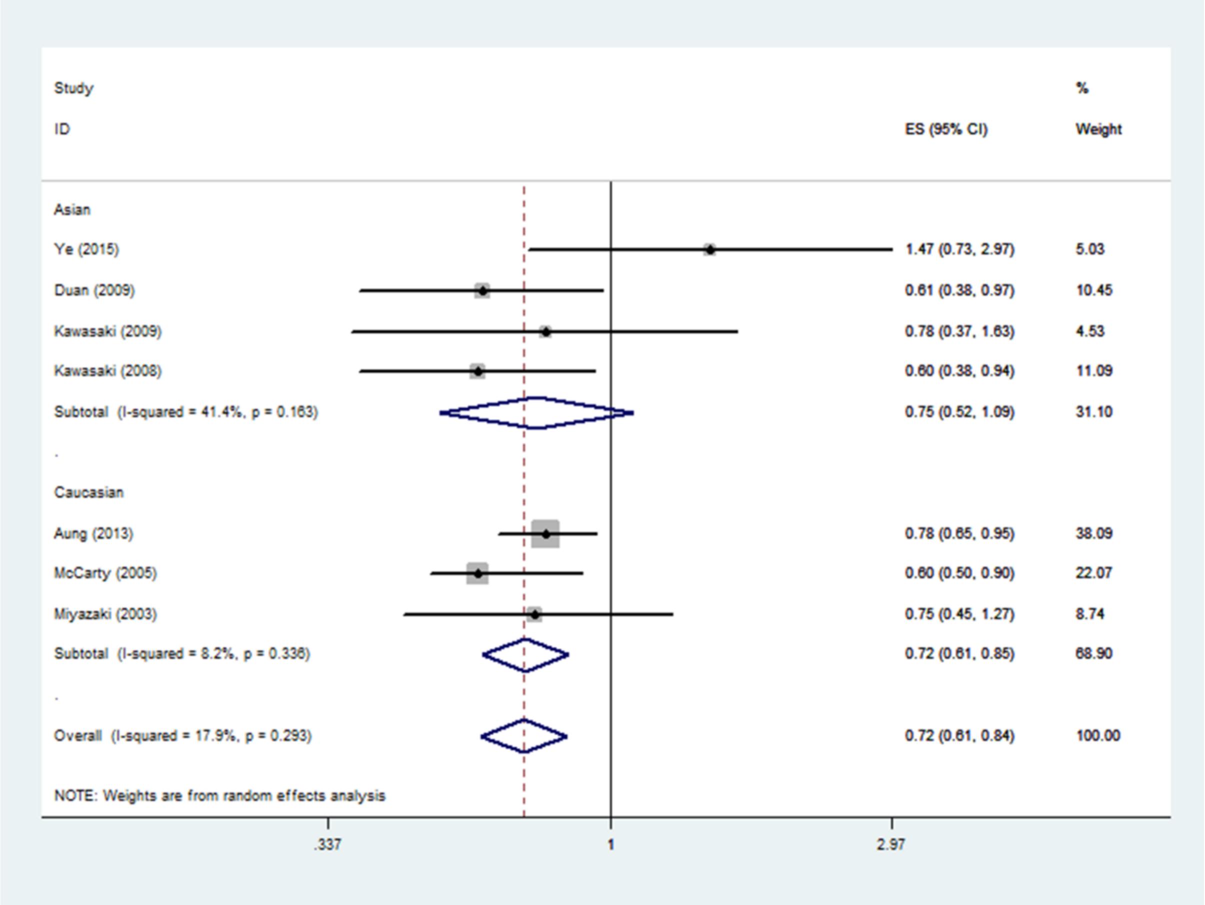
Study
ID
ES (95% CI)
%
Weight
Asian
Ye (2015)
1.47 (0.73, 2.97)
5.03
Duan (2009)
0.61 (0.38, 0.97)
10.45
Kawasaki (2009)
0.78 (0.37, 1.63)
4.53
Kawasaki (2008)
0.60 (0.38, 0.94)
11.09
Subtotal (I-squared = 41.4%, p = 0.163)
0.75 (0.52, 1.09)
31.10
Caucasian
Aung (2013)
0.78 (0.65, 0.95)
38.09
McCarty (2005)
0.60 (0.50, 0.90)
22.07
Miyazaki (2003)
0.75 (0.45, 1.27)
8.74
Subtotal (I-squared = 8.2%, p = 0.336)
0.72 (0.61, 0.85)
68.90
Overall (I-squared = 17.9%, p = 0.293)
0.72 (0.61, 0.84)
100.00
NOTE: Weights are from random effects analysis
.337
1
2.97

Figure S3

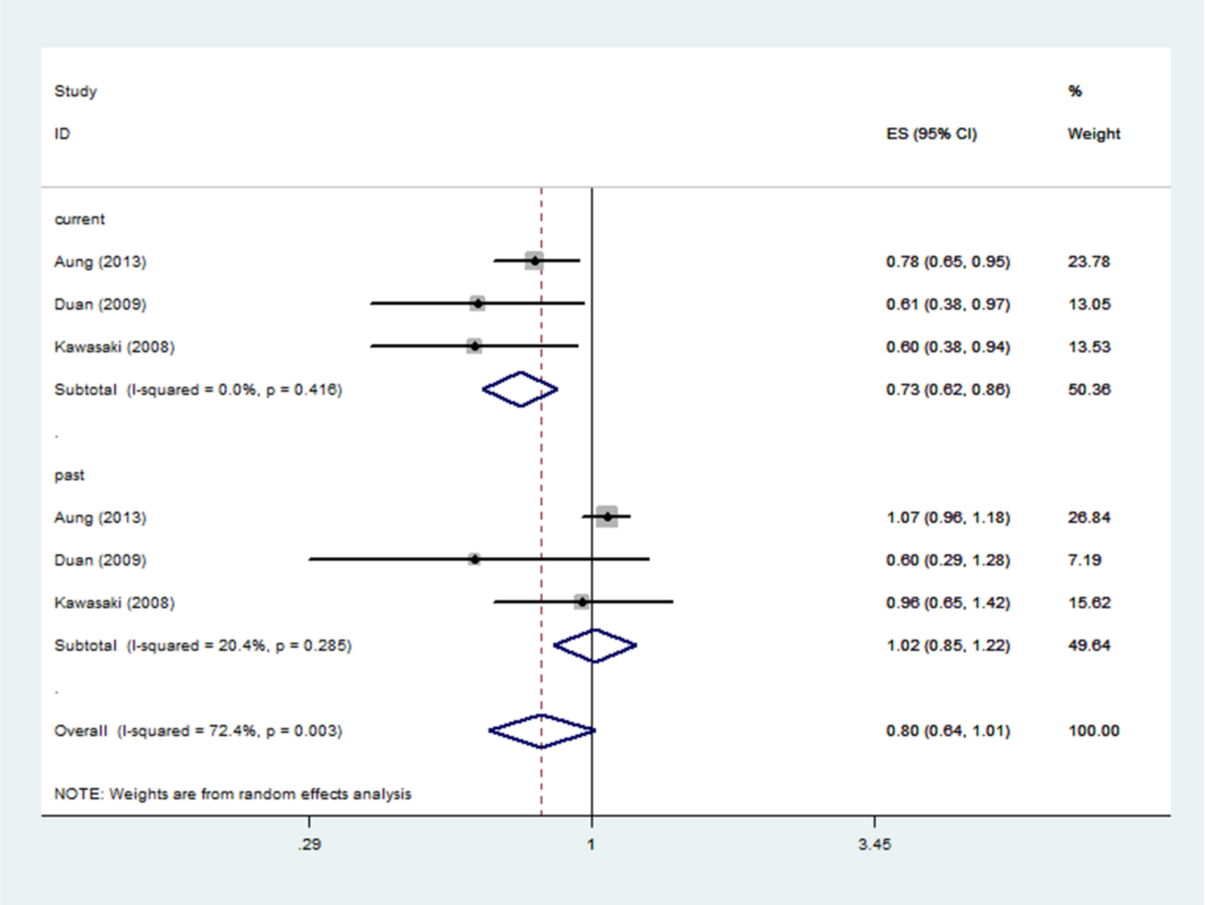

Study
ID
ES (95% CI)
%
Weight
current
Aung (2013) 0.78 (0.65, 0.95) 23.78
Duan (2009) 0.61 (0.38, 0.97) 13.05
Kawasaki (2008) 0.60 (0.38, 0.94) 13.53
Subtotal (I-squared = 0.0%, p = 0.416) 0.73 (0.62, 0.86) 50.36
past
Aung (2013) 1.07 (0.96, 1.18) 26.84
Duan (2009) 0.60 (0.29, 1.28) 7.19
Kawasaki (2008) 0.96 (0.65, 1.42) 15.62
Subtotal (I-squared = 20.4%, p = 0.285) 1.02 (0.85, 1.22) 49.64
Overall (I-squared = 72.4%, p = 0.003) 0.80 (0.64, 1.01) 100.00
NOTE: Weights are from random effects analysis
.29
1
3.45

Figure S4

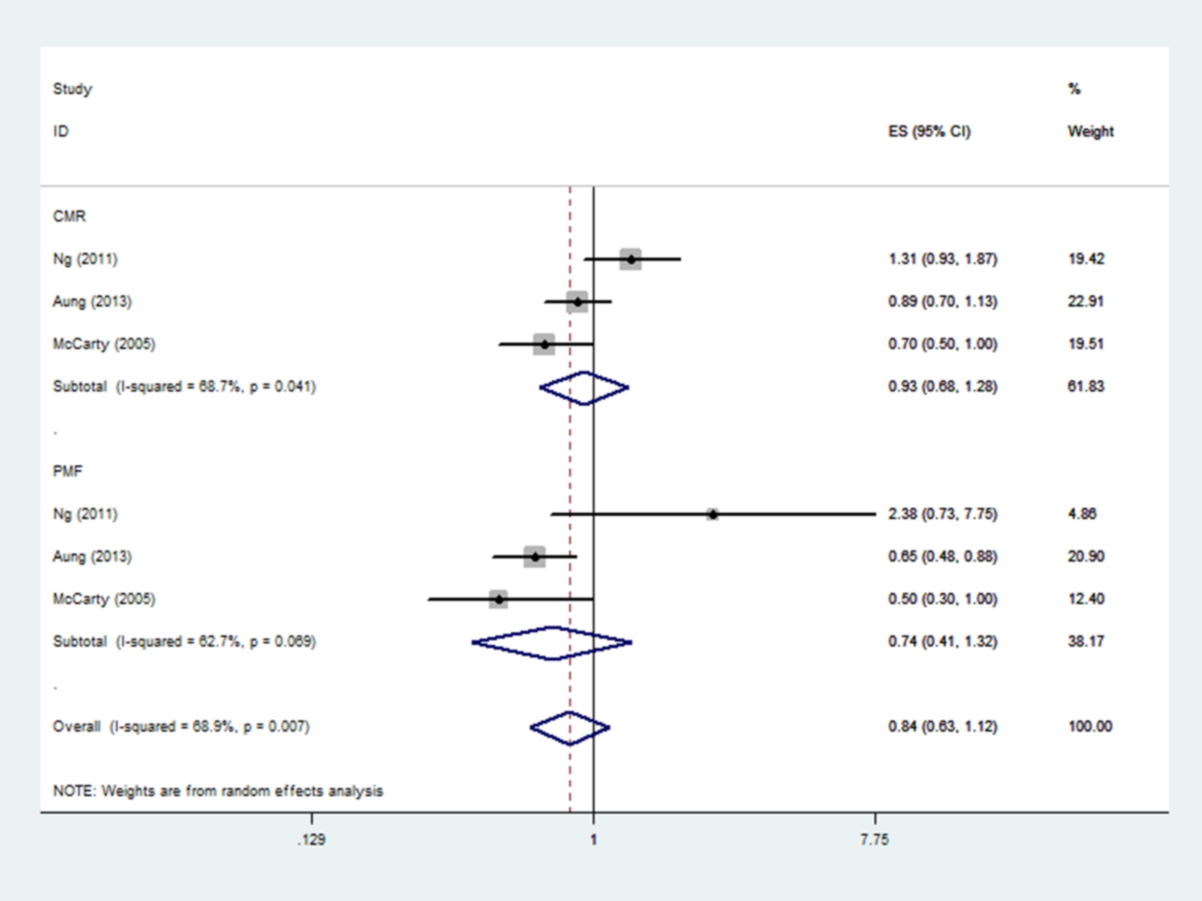

Study
ID
ES (95% CI)
%
Weight
CMR
Ng (2011) 1.31 (0.93, 1.87) 19.42
Aung (2013) 0.89 (0.70, 1.13) 22.91
McCarty (2005) 0.70 (0.50, 1.00) 19.51
Subtotal (I-squared = 68.7%, p = 0.041) 0.93 (0.68, 1.28) 61.83
PMF
Ng (2011) 2.38 (0.73, 7.75) 4.86
Aung (2013) 0.65 (0.48, 0.88) 20.90
McCarty (2005) 0.50 (0.30, 1.00) 12.40
Subtotal (I-squared = 62.7%, p = 0.069) 0.74 (0.41, 1.32) 38.17
Overall (I-squared = 68.9%, p = 0.007) 0.84 (0.63, 1.12) 100.00
NOTE: Weights are from random effects analysis
.129
1
7.75

Figure S5

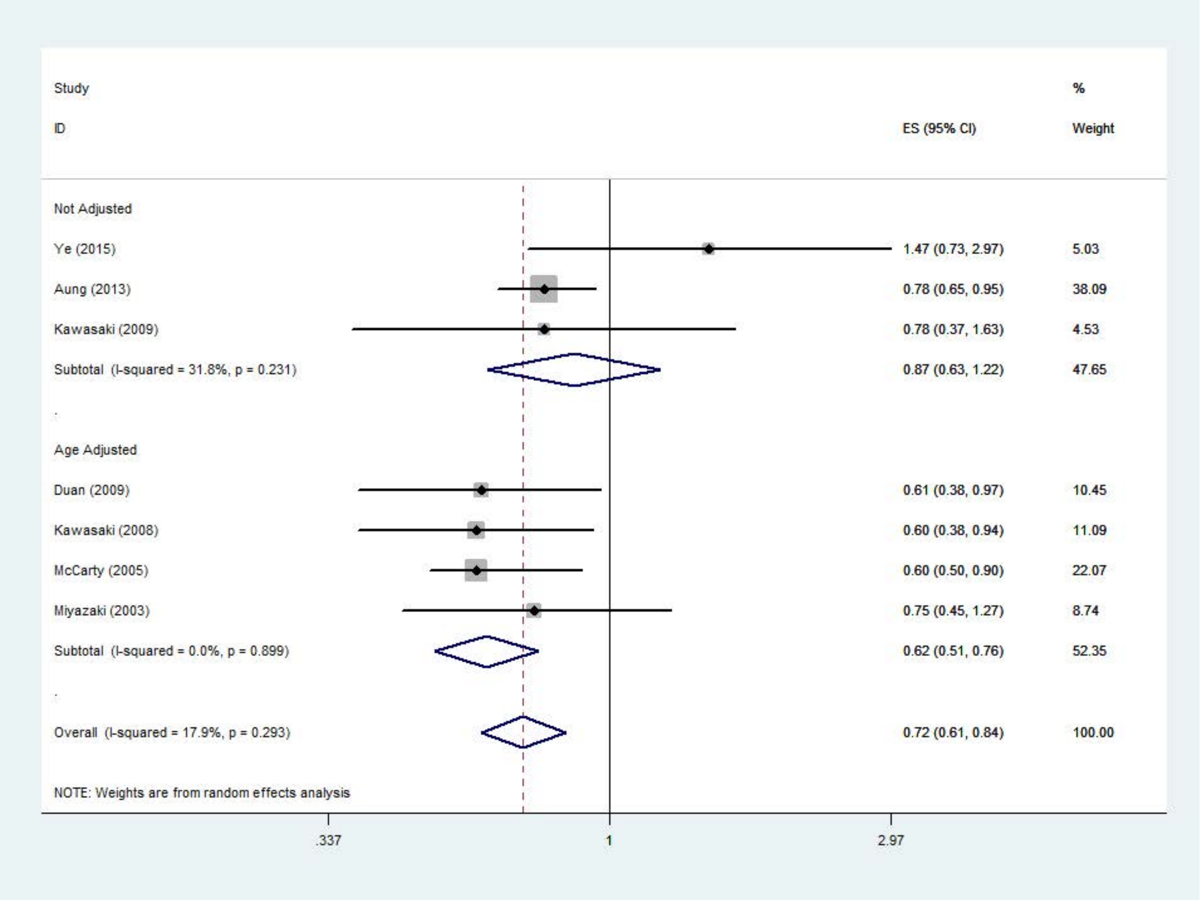

Study
ID
ES (95% CI)
%
Weight
Not Adjusted
Ye (2015)
1.47 (0.73, 2.97)
5.03
Aung (2013)
0.78 (0.65, 0.95)
38.09
Kawasaki (2009)
0.78 (0.37, 1.63)
4.53
Subtotal (I-squared = 31.8%, p = 0.231)
0.87 (0.63, 1.22)
47.65
Age Adjusted
Duan (2009)
0.61 (0.38, 0.97)
10.45
Kawasaki (2008)
0.60 (0.38, 0.94)
11.09
McCarty (2005)
0.60 (0.50, 0.90)
22.07
Miyazaki (2003)
0.75 (0.45, 1.27)
8.74
Subtotal (I-squared = 0.0%, p = 0.899)
0.62 (0.51, 0.76)
52.35
Overall (I-squared = 17.9%, p = 0.293)
0.72 (0.61, 0.84)
100.00
NOTE: Weights are from random effects analysis
.337
1
2.97

Figure Legend

Figure S1: Pooled estimates of association between smoking and epiretinal membrane in subgroup analysis based on study design. ES means odds ratio, CI, confidence interval.

Figure S2: Pooled estimates of association between smoking and epiretinal membrane in subgroup analysis based on population race. ES means odds ratio, CI, confidence interval.

Figure S3: Pooled estimates of association between smoking and epiretinal membrane in subgroup analysis based on smoking status. ES means odds ratio, CI, confidence interval.

Figure S4: Pooled estimates of association between smoking and epiretinal membrane in subgroup analysis based on epiretinal membrane classification. CMR, cellophane macular reflex, PMF, cellophane macular reflex, ES means odds ratio, CI, confidence interval.

Figure S5: Pooled estimates of association between smoking and epiretinal membrane in subgroup analysis based on age adjustment. ES means odds ratio, CI, confidence interval
